# Supplementary material for: Effects of Antithrombotic Treatment on Bleeding Complications of EBUS-TBNA
Source: Medicina (Kaunas). 2021 Feb 5;57(2):142. doi: 10.3390/medicina57020142 (PMC7916039; doi:10.3390/medicina57020142)
Supplement: Supplementary file 1 [file medicina-57-00142-s001.pdf]

Supplement Table 1. Baseline characteristics of patients who received EBUS-TBNA.

Variables	Insufficient discontinuation group (n = 102)	Sufficient discontinuation group plus no ATAs group (n = 4,169)	*p*
Age, years	69.7 ± 7.2	63.2 ± 11.1	<0.001
Sex, male	84 (82.4)	2,894 (69.4)	0.007
Pre-procedure diagnosis			0.436
Primary lung cancer	91 (89.2)	3,534 (84.8)	
Other cancer	8 (7.8)	434 (10.4)	
Other benign disease	3 (2.9)	201 (4.8)	
Laboratory tests			
Platelet count, ×10³/μL	252.6 ± 101.3	254.1 ± 81.8	0.881
Prothrombin time, INR	1.1 ± 0.3	1.0 ± 0.1	0.005
aPTT, seconds	37.7 ± 7.7	36.7 ± 5.4	0.223
Blood urea nitrogen, mg/dL	17.6 ± 7.2	15.6 ± 5.4	0.006
Creatinine, mg/dL	1.1 ± 0.7	0.9 ± 0.4	0.004
Total bilirubin, mg/dL	0.5 ± 0.3	0.5 ± 0.3	0.553
Aspartate aminotransferase, U/L	22.7 ± 12.2	22.2 ± 12.6	0.698
Alanine aminotransferase, U/L	20.2 ± 14.8	20.7 ± 16.3	0.750

Data are presented as number (percentage) or as mean ± standard deviation. EBUS-TBNA = endobronchial ultrasound-guided transbronchial needle aspiration; INR = international normalized ratio = aPTT, activated partial thromboplastin time.

Supplement Table 2. Characteristics of examined lesions.

Variables	Insufficient discontinuation group (n = 284)	Sufficient discontinuation group plus no ATAs group (n = 9,743)	*p*
Examined site			0.896
Mediastinal LN	231 (81.3)	7,875 (80.8)	
Hilar, interlobar, and lobar LN	42 (14.8)	1,434 (14.7)	
Other*	11 (3.9)	434 (4.5)	
Size of examined lesion, mm †			
Short-axis diameter	11.2 ± 6.7	11.7 ± 7.4	0.188
Long-axis diameter	16.2 ± 9.6	16.7 ± 10.4	0.369
Number of needle passes per lesion	1.9 ± 0.8	2.0 ± 1.0	0.009
Number of obtained core tissues per lesion	1.5 ± 0.7	1.6 ± 0.9	0.008
Procedure time, minutes	21.7 ± 12.1	19.9 ± 10.2	0.143

Data are presented as number (percentage) or as mean ± standard deviation. LN = lymph node.
* Lung parenchymal lesions and pleural seeding nodules were included. † Size on the transverse plane of computed tomography images.
